# Continuous non-invasive estimates of cerebral blood flow using electrocardiography signals: a feasibility study

**DOI:** 10.1007/s13534-023-00265-z

**Published:** 2023-02-09

**Authors:** Samuel J. van Bohemen, Jeffrey M. Rogers, Philip C. Boughton, Jillian L. Clarke, Joaquin T. Valderrama, Andre Z. Kyme

**Affiliations:** 1 School of Biomedical Engineering, The University of Sydney, Sydney, NSW Australia; 2 Neurocare Group, Sydney, NSW Australia; 3 Department of Clinical Medicine, Macquarie University, Sydney, NSW Australia; 4 Sydney Spine Institute, Sydney, NSW Australia; 5 Sydney Pharmacy School, Faculty of Medicine and Health, The University of Sydney, Sydney, NSW Australia; 6 Faculty of Medicine and Health, The University of Sydney, Sydney, NSW Australia; 7 National Acoustic Laboratories, Sydney, NSW Australia; 8 Linguistic Department, Macquarie University, Sydney, NSW Australia; 9 Brain and Mind Centre, The University of Sydney, Sydney, NSW Australia

**Keywords:** Cerebral blood flow, Continuous monitoring, Electrocardiography, Electroencephalography, Quantitative electroencephalography, Stroke

## Abstract

This paper describes a potential method to detect changes in cerebral blood flow (CBF) using electrocardiography (ECG) signals, measured across scalp electrodes with reference to the same signal across the chest—a metric we term the Electrocardiography Brain Perfusion index (EBPi). We investigated the feasibility of EBPi to monitor CBF changes in response to specific tasks. Twenty healthy volunteers wore a head-mounted device to monitor EBPi and electroencephalography (EEG) during tasks known to alter CBF. Transcranial Doppler (TCD) ultrasound measurements provided ground-truth estimates of CBF. Statistical analyses were applied to EBPi, TCD right middle cerebral artery blood flow velocity (rMCAv) and EEG relative Alpha (rAlpha) data to detect significant task-induced changes and correlations. Breath-holding and aerobic exercise induced highly significant increases in EBPi and TCD rMCAv (*p* < 0.01). Verbal fluency also increased both measures, however the increase was only significant for EBPi (*p* < 0.05). Hyperventilation induced a highly significant decrease in TCD rMCAv (*p* < 0.01) but EBPi was unchanged. Combining all tasks, EBPi exhibited a highly significant, weak positive correlation with TCD rMCAv (r = 0.27, *p* < 0.01) and the Pearson coefficient between EBPi and rAlpha was r = − 0.09 (*p* = 0.05). EBPi appears to be responsive to dynamic changes in CBF and, can enable practical, continuous monitoring. CBF is a key parameter of brain health and function but is not easily measured in a practical, continuous, non-invasive fashion. EBPi may have important clinical implications in this context for stroke monitoring and management. Additional studies are required to support this claim.

**Supplementary Information:**

The online version contains supplementary material available at 10.1007/s13534-023-00265-z.

## Introduction

Convenient and reliable continuous monitoring of cerebral blood flow (CBF) is challenging. Adequate CBF, nominally 50 mL/100 g/min [1], is vital for brain health and functions and is a key parameter for diagnosing and treating cerebrovascular diseases like acute ischaemic stroke (AIS). Computed tomography perfusion (CTP) imaging provides robust voxel-wise maps of CBF, however only at sparse time points that are typically days or weeks apart. More frequent CTP imaging is not practical because of the radiation dose associated with each acquisition. Thus, CTP imaging provides valuable ‘snapshots’ of CBF but does not provide continuous monitoring that could support real-time treatment monitoring and the detection of new stroke events.

Transcranial Doppler (TCD) ultrasound, functional near infrared spectroscopy (fNIRS), and rheoencephalography (REG) are non-invasive methods to continuously monitor CBF [2–4]. However, none have widespread clinical use in the context of stroke*.* TCD measures CBF velocity (CBFv) rather than flow rate. A linear relationship between the two is dependent on a constant vessel diameter [5]. TCD can be inaccurate in 5–20% of patients due to inadequate acoustic transtemporal bone windows and suffers from high intra-individual variability [3, 6]. The accuracy of fNIRS is influenced by spatial resolution, signal artefacts and lack of standardisation [7] thus is chiefly used as a research tool. REG monitors CBF by measuring the electrical conductivity between two scalp electrodes. It is inexpensive and can be wearable. However, REG is rarely used for clinical application due to the current lack of pathological and physiological correlations [2].

Electroencephalography (EEG) is a non-invasive method for the continuous monitoring of brain electrical activity. EEG is used to detect and describe both healthy brain function and neuropathology [8]. Although not a direct measure, EEG is also sensitive to changes in CBF due to related changes in metabolic and neural activity [1]. Currently, however, there are no reliable, wearable measurement systems for practical, continuous monitoring of CBF in stroke patients.

The heart generates the largest electrical signal in the body, typically measured using the electrocardiogram (ECG). And, as one of the most electrically conductive components of the body, blood provides a major pathway for the propagation of this electrical signal [9]. Given that a change in blood flow changes the electrical conductivity of the blood [10], we hypothesise that changes in CBF will manifest in the amplitude of the ECG signal recorded across scalp electrodes, with respect to the same signal recorded across the chest.

The aim of this study was to investigate the feasibility of this ECG-based measurement—termed the Electrocardiography Brain Perfusion index (EBPi)—to detect changes in CBF during tasks known to impact CBF, to characterise its sensitivity and performance, and to validate the findings against gold standard CBF measures.

## Methods

### Device construction, setup, and principle of operation

**Fig. 1:**
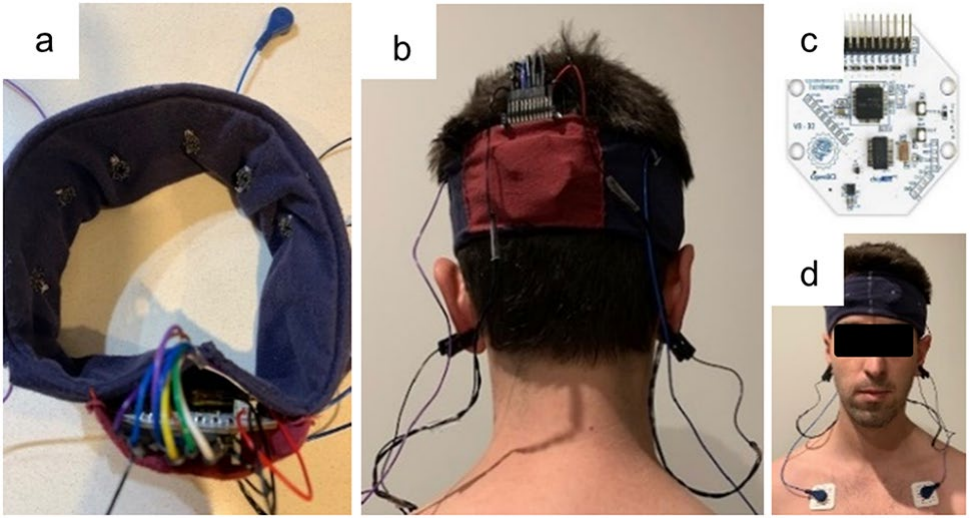
Prototype headset for ECG and EEG monitoring. A wearable EEG recording device (**a**, **b**) containing the OpenBCI Cyton Board (**c**) and adapted for ECG signal processing across the scalp and chest (**d**)

**Fig. 2:**
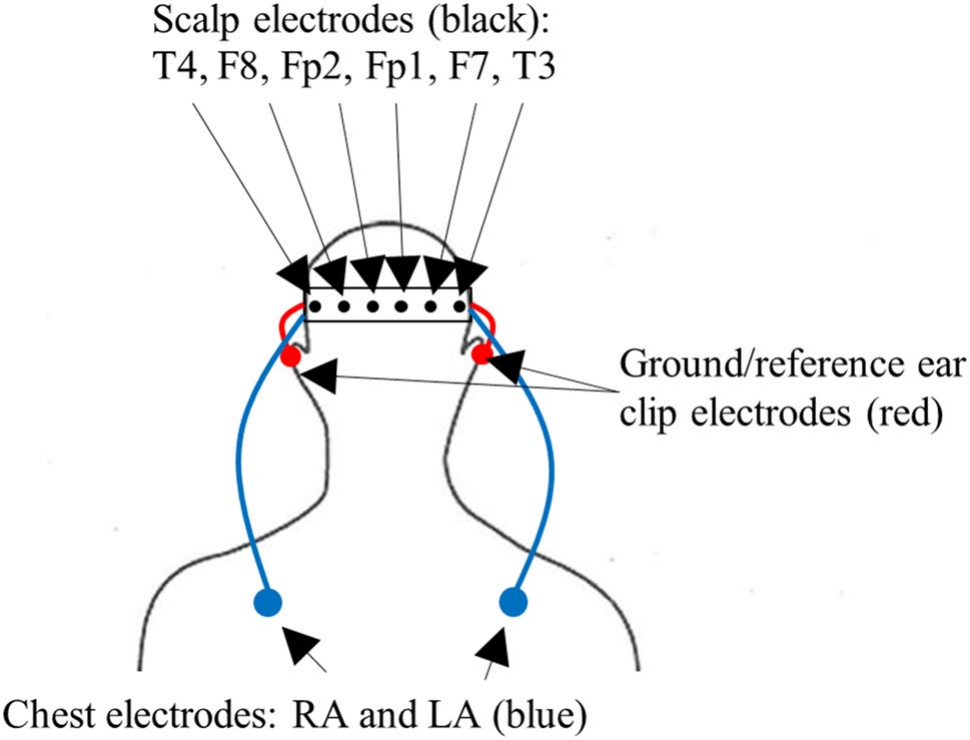
Device electrode layout

An EEG headset (Fig. 1) was constructed with an elastic fabric headband, an 8-channel 250 Hz sampling OpenBCI Cyton Board (Cyton Biosensing Board, OpenBCI), six EEG leads terminating in plastic Ag–AgCl coated electrodes, two ECG chest leads terminating in snap ECG electrodes, two ground/reference ear clip electrodes and a small lithium-ion battery (3.7 V, 400 mA). Electrode placement is shown in Fig. 2. The scalp electrodes (Fp1, Fp2, F7, F8, T3 and T4) were positioned according to the international 10–20 system, adjacent the frontal lobe, allowing access to the transtemporal bone window for TCD measurements of the middle cerebral artery (MCA). Two ECG chest leads (LA, RA Fig. 2) were attached below each clavicle using adhesive ECG electrodes. The ground/reference electrodes were placed on each ear lobe (Fig. 1b). The OpenBCI Cyton Board and battery were secured in a pocket on the back of the headband (Fig. 1a). Continuous EEG and ECG data were streamed wirelessly (OpenBCI RF USB dongle) to a nearby laptop during the experimental trials.

### Task choice

Breath-holding, hyperventilation, verbal fluency, and aerobic exercise were identified as tasks that induce changes in CBFv, which can be measured using TCD [11–14]. Breath-holding increases the partial pressure of CO_2_ (PaCO_2_) in the brain, leading to vasodilation of cerebral arterioles, increasing CBFv [13]. Hyperventilation decreases PaCO_2_ in the brain, leading to vasoconstriction of cerebral arterioles, decreasing CBFv [12]. Verbal fluency tasks involve letter-specific word retrieval, causing increased neural activation in the frontal and temporal lobes and a concomitant increase in CBFv [14]. Low and moderate intensity aerobic exercise increases CBFv, however this effect is reduced during vigorous exercise [11].

### Participants

Twenty healthy adult volunteers (mean age 32 yr, range 18–60 yr, SD 13.1 yr) participated in the study. Before testing, participants fasted for two hours and refrained from caffeine and vigorous exercise for 6 and 12 h, respectively [11]. Participants were to be 18 years or older. Participants with any history of epilepsy (n = 1) were excluded from the hyperventilation task. There were no other exclusion criteria.

### Experimental protocol

**Fig. 3:**
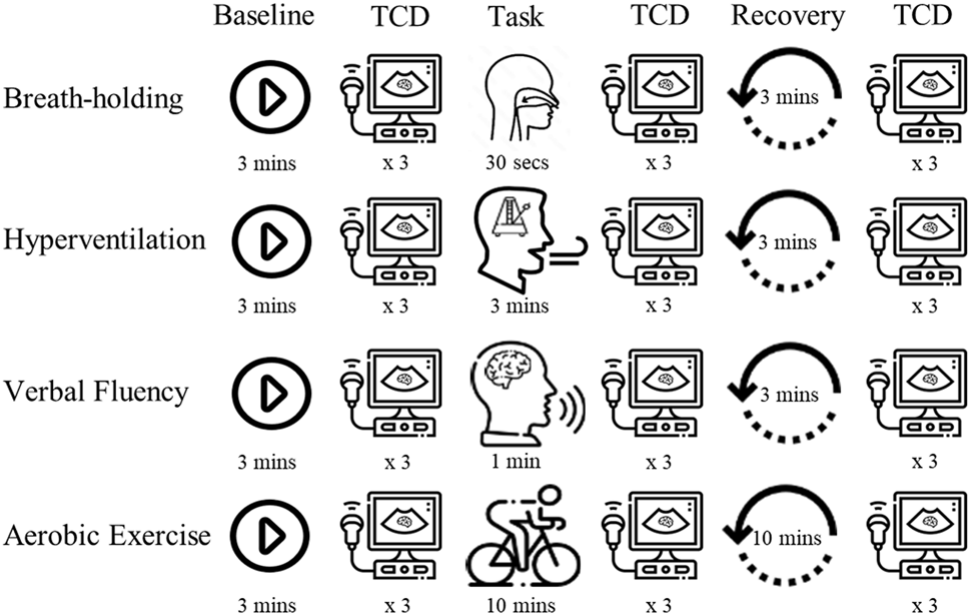
Study design. For each task (breath-holding, hyperventilation, verbal fluency, and aerobic exercise), ECG and EEG data were collected during baseline, task, and recovery for the times indicated. Triplicate TCD measurements were obtained immediately after each of these phases

All experimental procedures were conducted in accordance with an approved human ethics protocol (USyd 2019/1008). After the headset was fitted, data were collected according to Fig. 3. Each trial consisted of a 3-min baseline phase, where participants were at rest and relaxed, a time-on-task phase, and a recovery phase. The recovery phase was 3-min following the breath-holding, verbal fluency and hyperventilation tasks, and 10-min following aerobic exercise. All phases except for the aerobic exercise task were performed with eyes closed to avoid EEG eye-blink artefacts. After each phase, the right MCA (rMCA) was insonated to measure rMCA blood flow velocity (rMCAv) using a 1–5 MHz phased array transducer on a Philips iU22 ultrasound machine (Philips Ultrasound, Bothell, WA, USA) at an angle of 30 degrees and a depth of approximately 5 cm. This was repeated 3 times in quick succession (within 30-s) to provide a reliable average TCD rMCAv measurement.

Prior to every baseline phase, electrode contact, and data streaming were checked, and the ultrasound probe was placed over the right transtemporal bone window to locate the rMCA.

For the breath-holding task participants held their breath for 30-s [13]. For the hyperventilation task, participants breathed in and out at normal tidal volume for 3-min, in time with a metronome set to 30 beats per minute [12]. The verbal fluency task required participants to say as many words starting with the letter ‘t’ (excluding proper nouns) as possible in 1-min [14]. They were instructed to avoid words with the same prefix, e.g., ‘try’, ‘trying’. Audio recordings of the participants’ answers were used for scoring. For the aerobic exercise task, participants wore a chest strap (TICKR Heart Rate Monitor, Wahoo) to obtain a resting heart rate (rHR) and to monitor heart rate (HR). One participant wore a wrist-based HR monitor (Fenix 6x, Garmin). Moderate intensity exercise was defined as 50% of the heart rate reserve (HRR) calculated using the Karvonen formula and age-predicted maximum HR [11]:

1 $$50\% \, HRR = \frac{{\left( {220 - {\text{age}} + rHR} \right)}}{2}$$

Participants were seated on an exercise bike (Monark Ergomedic 828 E, Monark Sports and Medical) and instructed to pedal at 60 revolutions per minute (RPM). The RPM was displayed on a screen to provide feedback. Resistance started at 5 N and increased by 5 N each minute until the target HR (50% HRR) was reached. If the target HR was exceeded, the resistance was decreased by 2.5 N. All participants reached the target HR after 4–8 min. Post-exercise, participants recovered in a chair for 10-min. Preliminary testing indicated that this longer recovery time was necessary for CBF to return to baseline levels.

Participants chose which of the four tasks to perform. The same order of tasks was followed by all participants (breath-holding, hyperventilation, verbal fluency, aerobic exercise), skipping tasks that were not chosen (not all participants performed all tasks). Participants performing more than one task had a 10-min resting break between tasks to ensure CBF returned to baseline [15].

### Electrode contact

In several participants, good contact at T3 and T4 was not possible due to interference from hair, thus T3 and T4 were excluded from analysis in all participants.

### Data processing and analysis

#### Electrocardiography brain perfusion index (EBPi)

EBPi was implemented by capturing ECG data at each scalp electrode re-referenced to chest electrode LA (heart adjacent) (online supplementary information Fig. S1b). ECG data were also captured across the chest by re-referencing chest electrode RA to chest electrode LA (online supplementary information Fig. S1c).

EBPi was processed using a custom MATLAB (MathWorks Inc.) script. First, a fourth-order bandpass filter from 5–60 Hz was applied to the re-referenced data to filter noise. R-wave peaks and S-wave troughs of ECG QRS-complexes appearing in all of the re-referenced data (scalp electrodes and across the chest) were then detected using the MATLAB ‘findpeaks’ function. Non-outlier R-wave peaks and S-wave troughs were identified using the MATLAB ‘rmoutliers’ function. The amplitude difference between each R-wave peak and S-wave trough of every ECG QRS-complex at each scalp electrode was then computed relative to the amplitude difference between the R-wave peak and S-wave trough of the corresponding ECG QRS-complex across the chest. For each scalp electrode, EBPi was computed for a given time window ${\Delta }t$ according to:

2 $$EBPi_{{k, \Delta t_{j} }} = \frac{{\mathop \sum \nolimits_{i}^{{N_{{j}} }} \frac{{A_{k,i} }}{{A_{i}^{\prime } }}}}{{N_{{{j} }} }}$$

where $k$ indexes the electrode, ${\Delta }t_{j}$ denotes the *j*-th time interval which contains $N_{{{j} }}$ non-outlier QRS-complexes indexed by $i$, $A_{k,i}$ is the amplitude difference between the R-wave peak and S-wave trough of the $i$-th QRS-complex for scalp electrode $k$ measured with respect to chest electrode LA, and $A_{i}^{^{\prime}}$ is the amplitude difference between the R-wave peak and S-wave trough of the $i$-th QRS-complex for chest electrode RA measured with respect to chest electrode LA. Task-based ${\Delta }t$ values are shown in Table 1 and were chosen to ensure several ECG QRS-complexes were found in each window. For each experimental trial, EBPi values were normalised (offset) to the mean baseline value of the scalp electrode. We then computed mean EBPi during baseline, task, and recovery for each electrode. For each task grand average EBPi values for a specific scalp electrode were obtained by averaging the baseline-corrected values in (2) across all participants. In two participants the ECG data captured at RA was inverted, most likely due to the orientation of the heart axis. To process data consistently, RA data from these two participants were first inverted.

**Table 1:** Task-based time windows (Δ*t*) for EBPi calculation

Task	Time window (Δ*t*) (s)
Breath-holding	15
Hyperventilation	20
Verbal fluency	20
Aerobic exercise	30

#### EEG processing

EEG data were also processed using MATLAB. The data processing pipeline consisted of:

1. Noise filtering of the raw EEG data (fourth-order bandpass, 0.5–30 Hz);
2. Segmenting the data into 4-s epochs with 50% overlap, excluding any epochs containing values outside the range [− 100 µV, 100 µV];
3. Performing a Fast Fourier Transform on the epochs to extract the absolute power in the delta (1.5–3.5 Hz), theta (3.5–7.5 Hz), alpha (7.5–12.5 Hz) and beta (12.5–25 Hz) frequency bands;
4. Computing the relative power (%) of each frequency band with respect to the total absolute power across the four bands;
5. Computing continuous qEEG metrics for each scalp electrode using a 30-s window with 50% overlap for the duration of each trial;
6. Computing mean qEEG metrics for each scalp electrode based on the measures in (5), for each phase (baseline, task, and recovery) of each trial.

No eye-blink artefact suppression was applied since all EEG data were collected with eyes closed.

Decreased EEG alpha activity has been demonstrated during breath-holding, hyperventilation, and verbal fluency tasks, and following aerobic exercise [12, 14, 16, 17]. Therefore, relative alpha (rAlpha) was chosen as the qEEG measure to compare with EBPi.

Participants with any history of epilepsy (n = 1) were excluded from EEG processing.

A sample of EEG data captured during the study is presented in the online supplementary information Fig. S1a.

#### TCD data processing

For each trial, TCD rMCAv (cm.s^−1^) was calculated automatically according to:

3 $$rMCAv = \frac{{V_{psv} + 2V_{edv} }}{3}$$

where $V_{psv}$ and $V_{edv}$ denote peak systolic velocity and end diastolic velocity, respectively.

The three repeat measurements of TCD rMCAv captured at each phase (baseline, task, recovery) were averaged to produce mean TCD rMCAv for each experimental trial. Grand averages were also calculated by averaging mean baseline, task, and recovery TCD rMCAv across all participants for each task.

#### Statistical analysis

The Student’s paired t-test (two-tailed) was used to assess differences in baseline, task, and recovery phases for each of mean EBPi, TCD rMCAv and rAlpha separately, for all experimental trials and grand averages for all tasks. For EBPi and rAlpha this was computed for all electrodes. The Pearson r coefficient and the Student’s two-tailed t-test were used to assess the correlation between mean EBPi, TCD rMCAv and rAlpha for the task and recovery phase, using data from all 74 trials. Baseline-corrected values were used in all correlation analyses, and significance was taken to be *p* < 0.05.

## Results

### Participant demographics

Demographic and task participation data for the 20 volunteers (12 males, 8 females) are shown in Table 2. Seventy-four trials were completed in total.

**Table 2:** Volunteer demographics and task participation

Task	Male n (mean age (yr), SD)	Female n (mean age (yr), SD)	Combined n (mean age (yr), SD)
Breath-holding	12 (36.6, 14.3)	7 (24.6, 6.6)	19 (32.2, 13.2)
Hyperventilation	12 (36.6, 14.3)	7 (23.0, 6.1)	19 (31.6, 13.5)
Verbal fluency	11 (37.1, 14.8)	7 (24.6, 6.6)	18 (32.2, 13.6)
Aerobic exercise	12 (36.6, 14.3)	6 (23.0, 5.6)	18 (32.1, 13.6)

n: Number of participants; yr: Years; SD: Standard deviation

### Task-based data analysis

#### Breath-holding

**Fig. 4:**
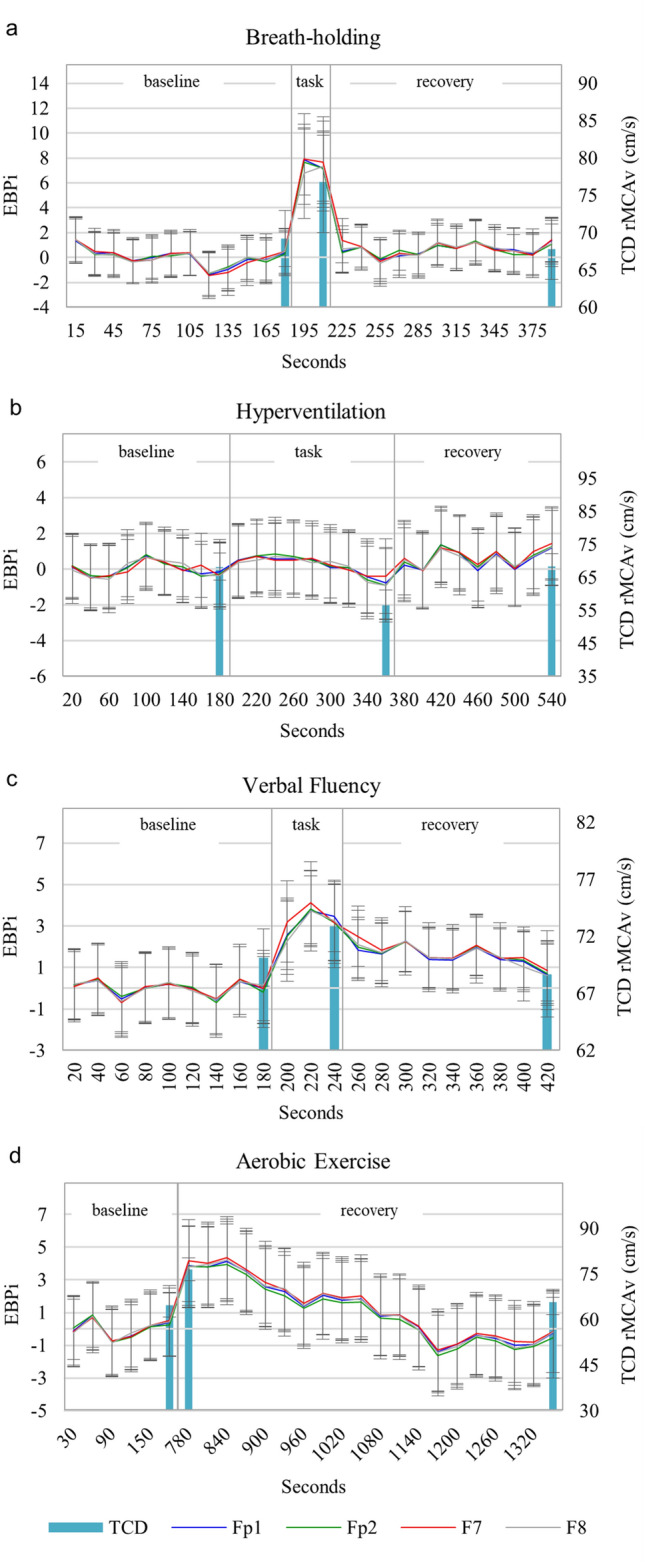
**a-d** Grand average EBPi and TCD rMCAv during **a** breath-holding, **b** hyperventilation, **c** verbal fluency and **d** aerobic exercise. Lines represent grand average EBPi recorded at scalp electrodes Fp1, Fp2, F7 and F8. Bars represent grand average TCD rMCAv. Error bars show ± 1 standard deviation. Streaming of the EEG and ECG data were interrupted during one breath-holding trial, resulting in a shortened baseline phase, and five aerobic exercise trials, resulting in a shortened task and recovery phase in one trial and a shortened recovery phase in four trials

**Fig. 5:**
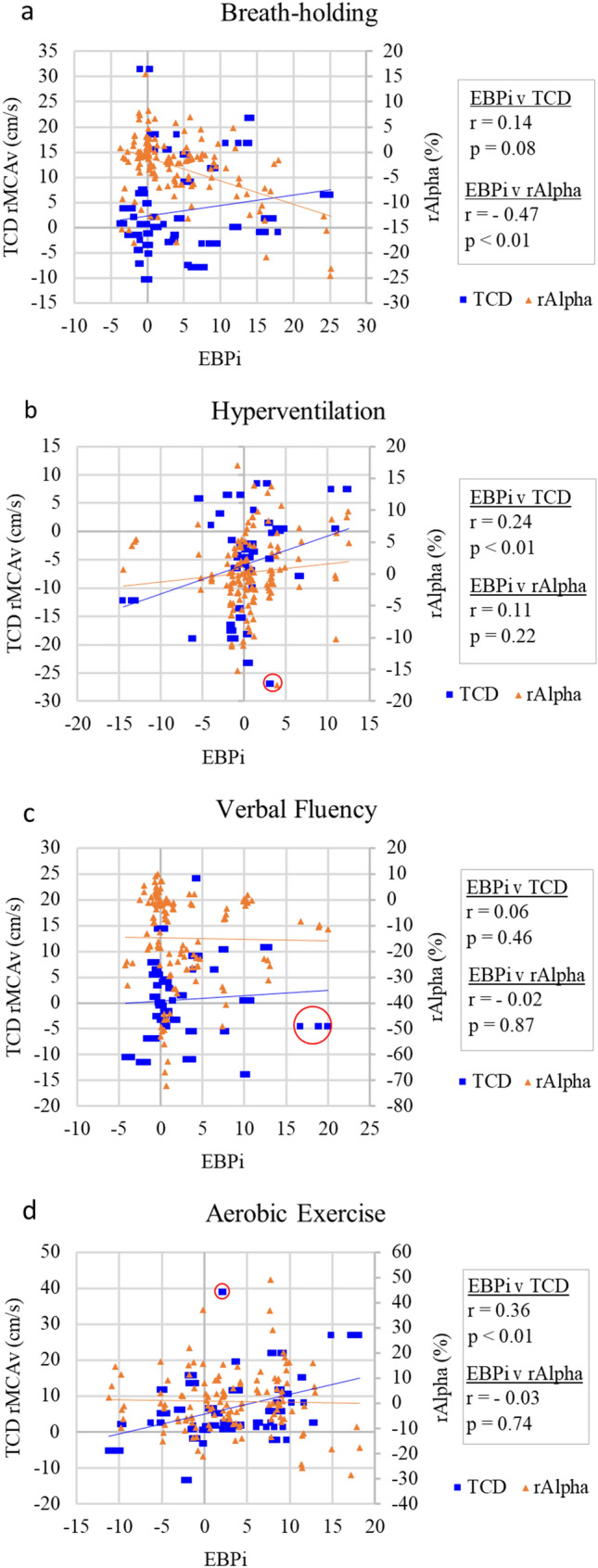
**a-d** Correlation between baseline-corrected mean EBPi, TCD rMCAv and rAlpha for the task and recovery phase of **a** breath-holding, **b** hyperventilation, **c** verbal fluency and **d** aerobic exercise. Outliers are circled in red

During the breath-holding task (Fig. 4a), grand average EBPi sharply increased from an electrode mean baseline value of 0 (± 1.8) to a mean task value of 7.4 (± 3.2). During the recovery phase grand average EBPi decreased to an electrode mean value of 0.6 (± 1.7). The increase from baseline-to-task and the decrease from task-to-recovery were both highly significant for all electrodes (*p* < 0.01). Mean TCD rMCAv also showed a highly significant increase following breath-holding and decrease following recovery (*p* < 0.01). Mean rAlpha significantly decreased following breath-holding and significantly increased following recovery in all electrodes (*p* < 0.05). The Pearson coefficient between EBPi and TCD rMCAv was r = 0.14 (*p* = 0.08) and EBPi was negatively correlated to rAlpha (r = − 0.47, *p* < 0.01) (Fig. 5a).

#### Hyperventilation

During the hyperventilation task (Fig. 4b), grand average EBPi was unchanged from an electrode mean baseline value of 0 (± 1.8) to a mean task value of 0.2 (± 2.0). During the recovery phase grand average EBPi remained unchanged at an electrode mean value of 0.6 (± 2.1). However, mean TCD rMCAv showed a highly significant decrease with hyperventilation and a highly significant increase following recovery (*p* < 0.01). Hyperventilation and recovery resulted in non-significant changes in mean rAlpha in all electrodes except for a significant increase for Fp2 alone post-recovery (*p* < 0.05). EBPi was positively correlated with TCD rMCAv (r = 0.24, *p* < 0.01). The Pearson coefficient between EBPi and rAlpha was r = 0.11 (*p* = 0.22) (Fig. 5b).

#### Verbal fluency

**Fig. 6:**
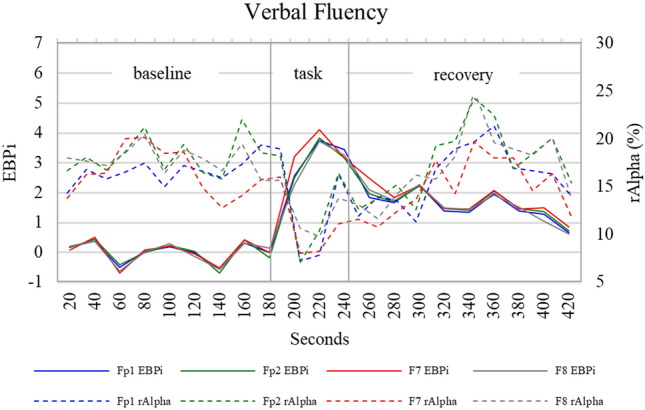
Grand average EBPi and rAlpha during verbal fluency at scalp electrodes Fp1, Fp2, F7 and F8. Error bars have been omitted for image clarity

During the verbal fluency task, participants named on average 14 words (range 5–28). During this task (Fig. 4c), grand average EBPi increased from an electrode mean baseline value of 0 (± 1.7) to a mean task value of 3.3 (± 1.9). During the recovery phase grand average EBPi decreased to an electrode mean value of 1.6 (± 1.5). The increase in mean EBPi from baseline-to-task was statistically significant in all electrodes (*p* < 0.05) but the decrease from task-to-recovery was not. Mean TCD rMCAv also increased following the task and decreased following the recovery phase, but neither was significant. The verbal fluency task caused a significant decrease in mean rAlpha in all electrodes (*p* < 0.05). Following the recovery phase there was a highly significant increase in mean rAlpha in all electrodes (*p* < 0.01). The Pearson coefficient between EBPi and TCD rMCAv was r = 0.06 (*p* = 0.46) and between EBPi and rAlpha was r = − 0.02 (*p* = 0.87) (Fig. 5c). For illustrative purposes, grand average EBPi and rAlpha during verbal fluency are displayed in Fig. 6. Verbal fluency induced a maximum decrease in rAlpha that preceded maximum EBPi by 20-s. During the recovery phase, EBPi and rAlpha decreased and increased, respectively, back towards baseline levels.

#### Aerobic exercise

During the aerobic exercise task, chest motion resulted in frequent corruption of the ECG QRS-complex reference signal. The benefit of applying a bandpass filter to extract reliable ECG QRS-complex signals was marginal. Therefore, the task phase was removed from analysis and instead mean EBPi and rAlpha estimates were computed for the task phase using the first 30-s of the recovery phase, and for the recovery phase using the last 30-s.

Following the aerobic exercise task (Fig. 4d), grand average EBPi increased from an electrode mean baseline value of 0 (± 2.0) to a mean task value of 3.9 (± 2.5). During the recovery phase grand average EBPi decreased to an electrode mean value of 1.4 (± 2.5). The increase in mean EBPi from baseline-to-task was highly significant in Fp2 and F7 (*p* < 0.01) and significant in Fp1 and F8 (*p* < 0.05), but the decrease from task-to-recovery was not significant in any electrode. Mean TCD rMCAv showed a highly significant increase from baseline-to-task and decrease from task-to-recovery (*p* < 0.01). Mean rAlpha exhibited non-significant changes in all electrodes from baseline-to-task and task-to recovery. EBPi was positively correlated to TCD rMCAv (r = 0.36, *p* < 0.01). The Pearson coefficient between EBPi and rAlpha was r = − 0.03 (*p* = 0.74) (Fig. 5d).

#### Tasks combined

Combining data from all four tasks (74 trials) revealed a highly significant, weak positive correlation between EBPi and TCD rMCAv (r = 0.27, *p* < 0.01). The Pearson coefficient between EBPi and rAlpha was r = − 0.09 (*p* = 0.05).

## Discussion

There is a long history of utilising ECG signals to assess cardiac haemodynamics [18]. However, utilising the ECG signal to infer blood flow in other parts of the body such as the brain has attracted much less attention. We investigated the feasibility of an ECG-based metric (EBPi) to detect changes in CBF. We developed a headset that provides continuous monitoring of EBPi and assessed the sensitivity of EBPi to detect changes in CBF during different tasks by validating against TCD measurements of CBFv.

By performing signal averaging over several minutes, low-amplitude (~ 10 μV) ECG signals can be detected at EEG scalp electrodes [19]. We are interested in real-time, continuous analysis of ECG signals at EEG scalp electrodes, thus averaging across lengthy timescales is not desirable. To reliably detect ECG signals across the scalp for real-time processing, we re-positioned the reference electrode to chest electrode LA (adjacent the heart); this resulted in ECG signals with QRS-complexes with R-wave peak—S-wave trough amplitude differences of ~ 500–600 μV measured at EEG scalp electrodes (online supplementary information Fig. S1b).

We were able to replicate previously reported changes in CBFv induced by the four tasks using TCD [11–14]. EBPi displayed the same changes as TCD rMCAv during breath-holding, verbal fluency and aerobic exercise—that is, EBPi and TCD rMCAv both increased during the task and decreased during recovery. These findings indicate that EBPi and TCD may be monitoring a similar underlying physiology. EBPi measures were recorded at scalp electrodes adjacent to the frontal lobe whereas TCD measurements were obtained from the rMCA. Both the frontal lobe and rMCA are supplied by the internal carotid artery (ICA), thus the tasks performed are expected to have induced changes in CBF in the ICA resulting in changes in the frontal lobe and rMCA.

Hyperventilation induced a highly significant decrease in TCD rMCAv (*p* < 0.01), although EBPi was unchanged. However, when individual trials were assessed (Student’s t-test, two-tailed), hyperventilation induced a significant decrease in EBPi in 7/19 trials and TCD rMCAv in 9/19 trials, revealing that these measures have a similar sensitivity to this task. Furthermore, there was a highly significant, weak positive correlation between EBPi and TCD rMCAv for hyperventilation (r = 0.24, *p* < 0.01). End tidal CO_2_ was not monitored in this study, thus we were unable to determine if all hyperventilation trials involved adequate effort to induce hyperventilation and a subsequent decrease in CBF. This may have increased the variability in the hyperventilation results.

The correlation strength between EBPi and TCD rMCAv varied across the four tasks (Fig. 5). However, following outlier removal (red circles Fig. 5), a significant, weak positive correlation between EBPi and TCD rMCAv was detected in three of the tasks: hyperventilation r = 0.32; verbal fluency r = 0.18; aerobic exercise r = 0.36 (*p* < 0.05). When all four tasks were combined (outliers removed) the correlation between EBPi and TCD rMCAv was r = 0.29 (*p* < 0.01). Outliers were detected using the Mahalanobis distance, where *p* < 0.001 [20].

We were also able to replicate previously reported changes in EEG rAlpha induced by the four tasks [12, 14, 16, 17]. Changes in rAlpha cannot be attributed to eye-blinks [21] since all measurements were obtained with eyes closed. Although other physiological artefacts (e.g., eye movement with eyes closed) may have affected EEG signals, additional artefact detection and removal methods were not implemented.

EBPi did not display any temporal or directional changes similar to rAlpha in any of the tasks. For example, during the verbal fluency task, rAlpha decreased to a minimum in Fp1, Fp2 and F7 20-s into the task phase whereas EBPi reached a maximum in all electrodes a further 20-s later (Fig. 6). A previous study also reported a sudden drop in alpha power that preceded the maximal CBFv measured by TCD during verbal fluency [14]. Thus, these findings suggest that EBPi is sensitive to a different underlying physiology than rAlpha. Future studies will compare EBPi with additional EEG frequency bands and qEEG metrics that are implicated in specific tasks and/or pathologies.

When data from all four tasks were combined, there was a highly significant, weak positive correlation between EBPi and TCD rMCAv (r = 0.27, *p* < 0.01); and the Pearson coefficient between EBPi and rAlpha was r = − 0.09 (*p* = 0.05). These provide further evidence that EBPi is more likely to monitor a similar physiological process to TCD (i.e., CBF) than rAlpha (i.e. neural electrical activity).

Our data are also suggestive of both localised and global changes. Hyperventilation induced changes in rAlpha that were not consistent across the four scalp electrodes. This could indicate sensitivity to localised changes in neural activity. By contrast, EBPi changes were consistent across all four scalp electrodes during all tasks, which may indicate that EBPi is sensitive to global changes – at least for the electrode placement used in this study. Previous studies have reported bilateral changes in CBFv during all four tasks [11, 14, 22], hence consistent changes in EBPi across the four scalp electrodes might be expected. The sensitivity of EBPi to localised changes will be explored in future work using a device with more scalp electrodes.

Although the study provides evidence that EBPi is correlated with TCD, there were several limitations that prevent us from concluding categorically that EBPi serves as a proxy for CBF. Firstly, without dedicated solutions to limit or remove motion-corrupted ECG signals, motion-induced artefacts prevented the analysis of EBPi during the aerobic exercise task. Adapting front-end signal acquisition to reduce artefacts and/or signal processing to remove artefacts will be important to reliably sample EBPi during tasks associated with increased chest motion.

Secondly, the TCD data were obtained by a single, accredited, experienced sonographer (JLC) for consistency. The system involved a conventional handheld probe, meaning it was not possible to obtain measurements accurately and consistently during tasks involving head movement (hyperventilation, aerobic exercise), and instead the triplicate TCD measurements were obtained immediately post-task. A Student’s paired t-test (two-tailed) found no significant differences between the sequential measures captured following breath-holding, verbal fluency or aerobic exercise. Although a significant difference was detected between the 2nd and 3rd sequential TCD measure following hyperventilation (*p* = 0.02), there was no significant difference detected between the 1st and 2nd (*p* = 0.66) or 1st and 3rd (*p* = 0.11) measurements. Therefore, none of these measurements were removed from the analysis. These results indicate that the recovery of CBFv following each task is unlikely to have impacted the results. Nevertheless, contemporaneous TCD and EBPi measurement should still be preferred to remove this potential limitation.

Thirdly, the duration of the tasks may have impacted the results. The shortest tasks, breath-holding (30-s) and verbal fluency (1-min), exhibited the lowest Pearson coefficient between EBPi and TCD rMCAv (r = 0.14, *p* = 0.08 and r = 0.06, *p* = 0.46, respectively) compared to hyperventilation (3-min) and aerobic exercise (10-min) (r = 0.24, *p* < 0.01 and r = 0.36, *p* < 0.01, respectively). Thus, it is possible that more data might lead to significant correlations. Future studies will involve monitoring over longer periods of time.

And fourthly, the EBPi and EEG data collected may have been impacted by noise resulting from poor connectivity and/or electrode movement, since dry, plastic Ag–AgCl coated EEG scalp electrodes (OpenBCI) were used without any adhesive to keep the electrodes in place. Future device iterations will implement small, gel, adhesive EEG scalp electrodes to minimise the impedance and prevent electrode movement.

There are several physiological variables that may influence EBPi*.* Although EBPi is an ECG-based measure and all four tasks induced an increase in heart rate, only three of the tasks induced an increase in EBPi. When all four tasks were combined, the Pearson coefficient between EBPi and heart rate was r = 0.08 (*p* < 0.01). This suggests that EBPi is not simply a proxy for heart rate.

Displacement of the heart can cause large changes in the ECG signal recorded across all body surfaces. EBPi is deliberately cast as a ratio (Eq. 2) in order for scalp-specific variation to be detectable despite intra-subject and inter-subject variation, such as changes in the position and shape of the heart. Nevertheless, further investigation is required to understand the impact that factors like displacement of the heart may have on EBPi.

We did not measure cardiac blood flow in this study and therefore cannot be sure how potential task-induced changes in cardiac blood flow may have affected ECG amplitude, CBF, and EBPi. However, as mentioned in the Methods Sect. (2.2 Task Choice), the tasks performed in this study are known to induce changes in CBFv that are independent of changes in cardiac blood flow, and which are caused by neurovascular coupling. Aerobic exercise is known to cause an increase in cardiac blood flow [23] and CBFv, but the specific relationship between cardiac blood flow and CBFv during aerobic exercise was beyond the scope of our study. Importantly, we were able to replicate previously reported changes in CBFv during all of the four tasks using TCD. Furthermore, the main new finding we emphasise from our study is that EBPi displayed the same changes as TCD rMCAv during all of the tasks except hyperventilation. This is suggestive of a relationship between EBPi and CBF but is certainly not conclusive. A more targeted study is required to investigate any specific relationship between ECG amplitude, cardiac blood flow, CBF and EBPi.

This study demonstrates that EBPi reflects changes related to TCD measures of CBFv. A linear relationship between CBF and CBFv is dependent on a constant cerebral blood vessel dimeter [5]. Cerebral blood vessel dimeter, which may have changed during specific tasks (via neurovascular coupling and/or cerebral autoregulation [24]), was not monitored during the study therefore we cannot be sure if tasks induced changes in vessel diameter and how this may have affected EBPi. An additional study is required to investigate the impact of changing cerebral blood vessel diameter to determine if EBPi measures CBF or CBFv.

In principle EBPi is attempting to measure something similar to REG (i.e., CBF via changes in underlying tissue conductivity), however it does so by using ECG signals. Future work looking at a direct comparison between the two methods is required to understand their relative advantages and limitations.

EBPi has potential application in the context of stroke. There is currently no device for the continuous monitoring of CBF and neural activity between CTP scans. During these time periods, standard care relies on neurological assessments, which can be subjective [25] and cannot be performed if the patient is sedated or unconscious. Ischaemic stroke accounts for 85% of all stroke cases [26] and is the result of a sudden restriction in CBF caused by a blood clot or atherosclerosis (the build-up of fat, cholesterol and other substances on the arterial wall). The sudden interruption of CBF starves neurons of oxygen leading to changes in neuronal electrical activity, neuronal cell death, loss of neuronal functioning and, consequently, the onset of clinical stroke symptoms. Successful AIS treatment involves the restoration of CBF using thrombolytic drugs such as tissue plasminogen activator (t-PA) or via mechanical thrombectomy [27]. A device that is able to continuously monitor stroke could add great value to stroke patient management.

In summary, this study provides evidence that EBPi is an easily obtained ECG-based measure that may be related to CBF, and which is distinct from rAlpha. Additional testing is required to further validate the feasibility and clinical utility of this method and device. Future studies will involve long term, continuous monitoring of stroke patients to determine if EBPi is able to monitor treatment outcome and detect significant changes from baseline in real time which might be indicative of salient clinical events. Previous reports have shown that qEEG measures, such as the delta/alpha ratio (DAR), can be used to detect stroke and assess stroke treatment [27]. Because EBPi uses EEG electrodes, our device is able to monitor neural electrical activity simultaneously. EBPi measures could therefore be combined with qEEG measures sensitive to stroke, such as DAR [27], to increase sensitivity to stroke and the impact of stroke treatment. A wearable device that can provide long-term, continuous monitoring of CBF and neural activity between CTP scans could aid treatment management and allow for faster detection of secondary stroke, leading to faster intervention and improved patient outcomes.

## Conclusion

This study presents a novel ECG-based metric called EBPi to monitor changes in CBF. EBPi had a highly significant, weak positive correlation with TCD-based measures of blood flow in the rMCA for a variety of tasks and can facilitate practical, continuous detection of changes. It therefore has potential utility in the long-term monitoring of stroke patients. Additional studies are required to support this claim.

## Supplementary Information

Below is the link to the electronic supplementary material.

Supplementary file1 (PDF 684 KB)

Supplementary file2 (PDF 455 KB)
