# Supplementary material for: Continuous non-invasive estimates of cerebral blood flow using electrocardiography signals: a feasibility study
Source: Biomed Eng Lett. 2023 Feb 9;13(2):185–95. doi: 10.1007/s13534-023-00265-z (PMC10130316; doi:10.1007/s13534-023-00265-z)
Supplement: Supplementary file 1 — Supplementary file1 (PDF 684 KB) [file 13534_2023_265_MOESM1_ESM.pdf]

Continuous Non-invasive Monitoring of Cerebral Blood Flow Using Electrocardiography Signals: A Feasibility Study

Samuel J. van Bohemen[1], Jeffrey M. Rogers[2,3], Philip C. Boughton[4,5], Jillian L. Clarke[6], Joaquin T. Valderrama[7,8], Andre Z. Kyme[1,9]

Samuel J. van Bohemen
svan2111@uni.sydney.edu.au

1. School of Biomedical Engineering, The University of Sydney, Sydney, New South Wales, Australia.
2. neurocare Group, Sydney, New South Wales, Australia.
3. Department of Clinical Medicine, Macquarie University, Sydney, New South Wales, Australia.
4. Sydney Spine Institute, Sydney, New South Wales, Australia.
5. Sydney Pharmacy School, Faculty of Medicine and Health, The University of Sydney, Sydney, New South Wales, Australia.
6. Faculty of Medicine and Health, The University of Sydney, Sydney, New South Wales, Australia.
7. National Acoustic Laboratories, Sydney, New South Wales, Australia.
8. Linguistic Department, Macquarie University, Sydney, New South Wales, Australia.
9. Brain and Mind Centre, The University of Sydney, Sydney, New South Wales, Australia.

Online supplementary information

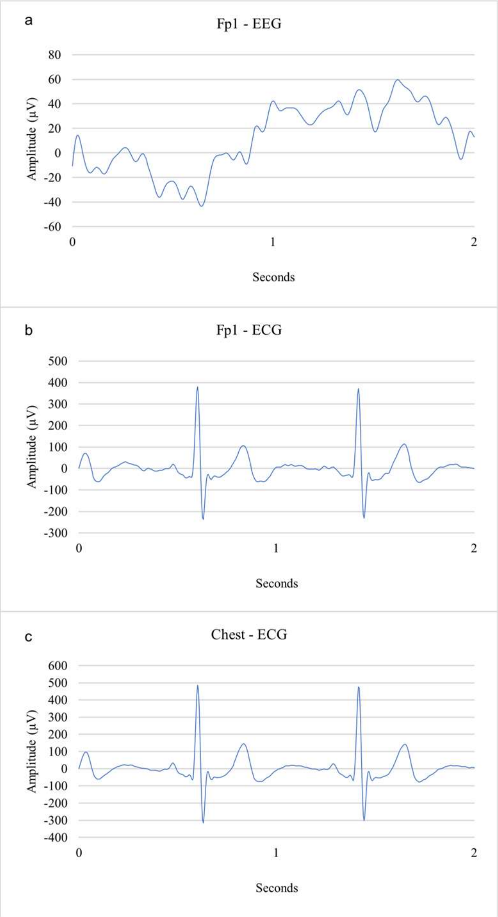

Fig. S1 EEG captured at scalp electrode Fp1 (a), re-referenced ECG QRS-complexes captured at scalp electrode Fp1 (b) and across the chest (c). These waveforms were captured at the same time from the same participant during the baseline period that preceded the breath-holding task.
