# Supplementary material for: Continuous non-invasive estimates of cerebral blood flow using electrocardiography signals: a feasibility study
Source: Biomed Eng Lett. 2023 Feb 9;13(2):185–95. doi: 10.1007/s13534-023-00265-z (PMC10130316; doi:10.1007/s13534-023-00265-z)
Supplement: Supplementary file 2 — Supplementary file2 (PDF 455 KB) [file 13534_2023_265_MOESM2_ESM.pdf]

Neuroergonomics Conference 2021

Conference Abstract

Continuous Monitoring of Cerebral Blood Flow in Healthy Volunteers and Stroke Patients

Samuel van Bohemen[1], Jeffrey Rogers[2], Jillian Clarke[2], Joaquin Valderrama[3,4] Philip Boughton[5,6,7], Andre Kyme[1,8]

[1]School of Biomedical Engineering, University of Sydney, Australia

[2]School of Health Sciences, University of Sydney, Australia

[3]National Acoustic Laboratories, Sydney, Australia

[4]Linguistic Department, Macquarie University, Sydney, Australia

[5]Sydney Spine Institute, Australia

[6]School of Pharmacy, Faculty of Medicine and Health, University of Sydney, Australia

[7]Charles Perkins Centre, Faculty of Medicine and Health, University of Sydney, Australia

[8]Brain and Mind Centre, University of Sydney, Australia

Background

Cerebral blood flow (CBF) is a key diagnostic parameter in acute ischemic stroke (AIS). Successful AIS treatment involves the restoration of CBF, for example via thrombolytic drugs like tissue plasminogen activator (tPA) [1]. The heart generates the largest electrical signal in the body and blood is one of the most electrically conductive components of the body, hence providing a major pathway for electrical signal propagation. When blood flow increases, the electrical conductivity of the blood also increases [2]. We hypothesise that increased CBF will increase conductivity of the blood and thus increase the amplitude of the electrocardiogram (ECG) recorded across scalp electrodes, with reference to the same signal recorded across the chest. This method may be able to monitor changes in CBF.

Objective

We have developed a new method to monitor changes in CBF using electroencephalography (EEG) and ECG signals and investigated the feasibility of this method to detect changes in CBF caused by hyperventilation. The results were validated against changes in CBF detected using transcranial Doppler (TCD) ultrasound.

Methods

Experimental Protocol

Hyperventilation induces a decrease in partial pressure CO_2 ($PaCO_2$) leading to vasoconstriction of cerebral arterioles and a decrease in CBF. Hyperventilation also causes a decrease in EEG alpha power [3]. For each of 6 participants, an EEG headband (OpenBCI 8-channel Cyton Board) with 4 scalp electrodes (Fp1, Fp2, F7, F8) and 2 chest leads (LA and RA) was fitted to the head. A ground/reference electrode was placed on the ear lobe. ECG electrodes were placed below each clavicle and the chest leads attached. EEG and ECG data were streamed continuously to a laptop during the experimental trials (see below). Our method was implemented by capturing ECG data at scalp electrodes by referencing each scalp electrode to chest electrode LA. ECG data were captured across the chest by referencing chest electrode RA to chest electrode LA. Spaced TCD measurements from the right middle cerebral artery

(rMCA) were also collected at 3 time points (immediately pre-task, immediately post-task, recovery) using a 1-5MHz sector ultrasound probe (Philips iU22) placed over the right transtemporal bone window.

Each experimental trial started with a 3-minute baseline period during which participants were relaxed with their eyes closed. Following the baseline period, 3 sequential measures of right middle cerebral artery blood flow velocity (rMCAv) were obtained using TCD. The participants were then instructed to breath in and out at normal depth in time with a metronome set to 30 beats per minute [3]. Immediately following the hyperventilation task, 3 more rMCAv TCD measurements were obtained. This was followed by a 3-min recovery period after which we collected the final 3 rMCAv TCD measurements.

Data Processing

A 4th-order bandpass filter 5-60 Hz was applied to the re-referenced ECG data. The amplitude of ECG complexes at scalp electrodes was divided by the amplitude of the ECG complexes across the chest to generate a metric called the Electrical Brain Perfusion Index (EBPi). EBPi values were then adjusted by the baseline mean. For EEG data, a 4th-order bandpass filter 0.5-30 Hz was applied. Epochs containing amplitudes +/- 100 uV were excluded from analysis. 4-second epochs (50% overlap) were transformed using the Fast Fourier Transform (FFT) with Hamming window to extract the absolute power in the following frequency bands: delta (1.5-3.5 Hz), theta (3.5-7.5 Hz), alpha (7.5-12.5 Hz), and beta (12.5-25 Hz). Relative power of each frequency band was calculated by summing the absolute power across the four bands to compute total power, and then dividing the absolute power of each frequency band by the total power, expressed as a percentage. The repeat TCD measures of rMCAv captured at 3 time points were averaged to produce a mean rMCAv for each phase.

Results

Figure 1 shows that the EBPi steadily decreased during the hyperventilation task, reaching a minimum at 320-340 seconds. During the first 20 seconds of the recovery period EBPi increased from -1.2 (+/-0.7) to 0.2 (+/- 0.6) and remained between 0 and 0.6 for the rest of the trial. EBPi values and changes were consistent across all electrodes. Mean EBPi significantly decreased ($p<0.05$, two-tailed t-test) from baseline to hyperventilation and increased from hyperventilation to recovery, in all electrodes. However, this increase was not significant ($p>0.05$, two-tailed t-test). TCD measurements of rMCAv (red boxes in Figure 1) significantly decreased ($p<0.05$, two-tailed t-test) from baseline following the hyperventilation task. Following the recovery period, rMCAv exhibited a clear return to baseline ($p<0.05$, two-tailed t-test). Hyperventilation caused a non-significant decrease ($p>0.05$, two-tailed t-test) in rAlpha power in electrodes Fp1 and Fp2 and a non-significant increase in electrodes F7 and F8 ($p>0.05$, two-tailed t-test). There was a large positive correlation between mean EBPi and TCD measures of rMCAv ($r=0.62$). This correlation was statistically significant ($p<0.05$, two-tailed t-test). There was also a non-significant positive correlation between mean EBPi and mean EEG rAlpha ($r=0.15$, $p>0.05$, two-tailed t-test).

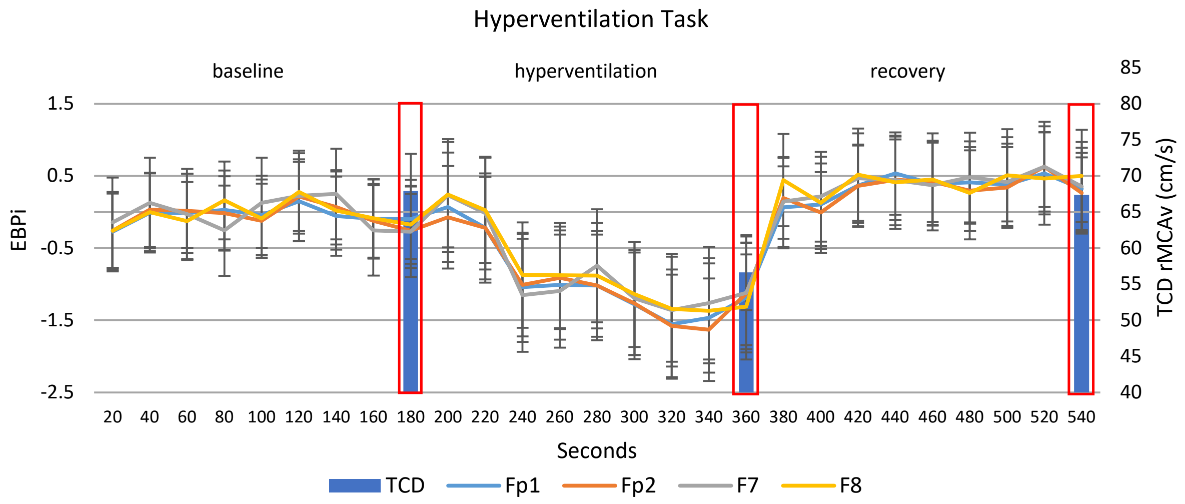

Figure 1. Electrical Brain Perfusion Index (EBPi) and transcranial Doppler (TCD) ultrasound right middle cerebral artery blood flow velocity (rMCAv) during a hyperventilation task (averaged across 6 participants). Line graphs represent EBPi recorded at scalp electrodes Fp1, Fp2, F7 and F8. Bar graphs represent mean TCD rMCAv. Red boxes represent the time windows during which TCD measures were obtained. Error bars = +/- 1 standard deviation.

Conclusion

Hyperventilation is known to cause a reduction in CBF, which we were able to detect using TCD measurements. We also demonstrated decreased EEG alpha power as per previous reports [3]. The novel EBPi measure showed a similar trend to the TCD measure of rMCAv and was significantly correlated to it. Conversely, the EBPi measure had no significant correlation with EEG rAlpha power. These results suggest EBPi measures a different physiological phenomenon than rAlpha power. The EBPi results for hyperventilation indicate that this measure may also be able to monitor changes in CBF induced by other factors. We are currently testing other tasks such as breath holding, verbal fluency and aerobic exercise. Since there are currently no devices for the continuous monitoring of stroke patients in-between CT scans, the method described could potentially fill this gap, thereby helping to inform treatment and detect secondary events.

Disclaimer

Samuel van Bohemen is the founder of Nuroflux Pty Ltd. Nuroflux Pty Ltd is the applicant of a filed provisional patent and PCT application (patent pending) for the described method of monitoring changes in CBF. Samuel van Bohemen, Philip Boughton and Andre Kyme are listed as inventors on the filed patent applications.

References

[1] S.-H. Lee, Stroke Revisited: Diagnosis and Treatment of Ischemic Stroke, Springer eBooks, 2017.

[2] A.E. Hoetink, T.J.C. Faes, K.R. Visser, and R.M. Heethaar, On the flow dependency of the electrical conductivity of blood. Ieee T Bio-Med Eng 51 (2004) 1251-1261.

[3] V. Kraaier, A.C. Vanhuffelen, and G.H. Wieneke, Changes in Quantitative EEG and Blood-Flow Velocity Due to Standardized Hyperventilation - a Model of Transient Ischemia in Young Human Subjects. Electroen Clin Neuro 70 (1988) 377-387.
